# Olfactory imagery as a retrieval method for autobiographical memories

**DOI:** 10.1007/s00426-022-01701-y

**Published:** 2022-07-05

**Authors:** Carina Schlintl, Saša Zorjan, Anne Schienle

**Affiliations:** 1 Clinical Psychology, University of Graz, BioTechMed, Universitätsplatz 2/III, 8010 Graz, Austria; 2 Department of Psychology, Faculty of Arts, University of Maribor, Slomškov trg 15, 2000 Maribor, Slovenia

## Abstract

**Introduction:**

The retrieval of autobiographical memories is influenced by several factors, such as sensory modality and the emotional salience of memory cues. This study aimed at investigating the interaction between sensory modalities (olfaction, vision) and emotional dimensions (valence, arousal) of imagery cues, on the frequency, quality, and age distribution of the autobiographical memories (AMs) elicited.

**Method:**

A total of 296 females (aged between 18 and 35 years) received one out of eight brief instructions for olfactory or visual imagery. The participants were asked to create a mental image with either high arousal/positive valence, high arousal/negative valence, low arousal/positive valence, or low arousal/negative valence (e.g., ‘imagine an unpleasant and arousing odor/scene’); no specific stimulus was mentioned in the instruction.

**Results:**

The approach used elicited imagery with autobiographical content in the majority of participants (78%). In terms of frequency, odor imagery, compared to visual imagery, turned out to be more effective at retrieving either unpleasant memories associated with experiences in adulthood, or pleasant childhood memories. In terms of quality, the imagery was rated as less vivid in the olfactory compared to the visual condition (irrespective of valence and arousal of the imagery instruction). Visual imagery was associated with the experience of more diverse emotions (happiness, sadness, anxiety, anger) than odor imagery, which was related primarily with disgust and happiness.

**Conclusion:**

Our findings indicate that nonspecific imagery induction is a useful approach in accessing AM.

**Implication:**

This approach presents promising clinical applications, such as in working with autobiographical memory narratives in psychotherapy.

**Supplementary Information:**

The online version contains supplementary material available at 10.1007/s00426-022-01701-y.

## Introduction

Random odors can evoke autobiographical memories (AMs; i.e., memories of personally experienced events); this is otherwise known as the Proust phenomenon. For instance, a certain odor may transport one suddenly back into the arms of one’s grandmother, who had used a perfume with a similar scent, or alternatively, it might remind another person of an unpleasant experience at the dentist’s.

The phenomenon of odors as retrieval cues for AMs has been the subject of various studies (see review articles: Chu & Downes, 2000; Hackländer et al., 2019; Herz, 2016; Larsson et al., 2014; Saive et al., 2014). Importantly, these experiments have shown that olfactory stimuli can elicit specific AMs that differ from the AMs evoked by other senses (e.g., images, sounds). For example, it was found that odors are effective at evoking early childhood memories (age < 10 years; Chu & Downes, 2000; Koppel & Berntsen, 2015; Willander & Larsson, 2006). Additionally, odor-evoked memories are generally rated as more emotional and vivid, compared to memories elicited by other sensory modalities (Chu & Downes, 2000; Willander & Larsson, 2007). At the same time, fewer AMs are reported following odor cues than after verbal or visual cues (e.g., Chu & Downes, 2000; Ernst et al., 2020; Willander & Larsson, 2007).

The present experiment expanded on previous findings concerning the effects of sensory cues on AM retrieval, and investigated whether olfactory imagery may present an alternative for accessing AMs. Imagery is generally defined as a mental representation in the absence of an external stimulus (Freeman, 1981). Mental images arise from neural activity in early sensory cortices (e.g., primary olfactory cortex [i.e., the piriform cortex]; Djordjevic et al., 2005). Indeed, a wide range of research has demonstrated that brain areas activated during odor imagery and perception overlap (e.g., Araujo et al., 2005; Bensafi et al., 2003; Djordjevic et al., 2004, 2005; Stevenson & Case, 2005). Hence, imagery might present itself as being an easy-to-use alternative to the application of specific external stimuli (e.g., odors, pictures) to trigger AMs, since no additional material is needed.

To the best of our knowledge, there has only been one study which has investigated olfactory imagery as a memory cue (Willander & Larsson, 2008). In that study, participants were randomized into one of two cueing conditions, the “verbal cue only” or the “verbal cue plus olfactory imagery” condition. In the “verbal cue only” condition, printed words were presented to the participants (e.g., tobacco, lily). In the “verbal cue plus olfactory imagery condition”, participants were also instructed to imagine the odor pertaining to the word. In the next step, participants were asked to describe and rate AMs which they associated with the cue. The results showed that mean AM retrieval rate was around 40%. Memories retrieved in the “verbal cue plus olfactory imagery” condition were older (i.e., memories from childhood) than memories evoked in the “verbal cue only” condition. Furthermore, memories evoked after olfactory imagery included higher proportions of sensory experiences, such as visual, olfactory, gustatory, or auditory sensations, as compared to the other condition. It is important to note, that the classical deliberate cueing method used in that study, which names specific cues (e.g., an odor of a lily) and asks the participants to retrieve an AM for these cues, can have several disadvantages. First, the results could have been affected by individual differences relating to experience, or lack of experience, with a certain cue (e.g., has a participant smelled a lily before?). Second, the emotional response to an odor is likely to be rated differently depending on a person’s previous experience with that odor.

Humans tend to immediately classify stimuli (of varying sensory modalities) in terms of the emotional dimensions valence and arousal (e.g., odors: Bensafi et al., 2002; pictures: Lang et al., 1997). Valence is defined as the degree of favorable feelings a person feels (in relation to given circumstances or to certain stimuli), and arousal is defined as the degree of excitement one feels (Lang, 1995; Russell, 1980). For example, the primary reaction to odors is either’liking’ or’disliking’; in other words, either a positive or negative valence (e.g. Ehrlichman & Halpern, 1988). This classification of sensory stimuli based on emotional dimensions (e.g., Ehrlichman & Bastone, 1992; Lang et al., 1997) can influence cognition, including memory retrieval. For example, Schulkind and Woldorf (2005), as well as Sheldon and Donahue (2017), found that the valence of the retrieved memories matches the valence of the retrieval cue (in this case, music), while at the same time, no match for arousal (between the memories evoked and the retrieval cue) was found. With regard to odors, for example, Ehrlichman & Halpern (1988) showed that subjects retrieved a significantly greater percentage of happy memories (which were evoked by a neutral word) after having perceived a pleasant odor, compared to subjects who had perceived an unpleasant odor.

Previous research has shown that there is a general bias to access positive memories (e.g. review: Walker et al., 2003) and arousing memories (e.g., Finkenauer et al., 1998; Talarico et al., 2004). However, recent empirical evidence suggests that cues of different sensory modalities may differ in relation to the emotional valence of retrieved memories. Ernst et al. (2020) showed that in contrast to other sensory modalities, odors elicited less positive memories, and more neutral and negative memories. Further, Knez et al. (2017) found interaction effects between sensory modalities and emotional dimensions of external cues on AM retrieval. In that study, unimodal visual (i.e., pictures), auditory (i.e., sounds), and olfactory stimuli (i.e., odors), with neutral (e.g., lighting of a match), positive (e.g., a campfire), and negative valence (e.g., violent fire), were used to induce AMs. It was found that pleasant visual stimuli (compared to auditory/olfactory cues) were superior at inducing positively valenced AMs, whereas aversive odors were effective at eliciting negative AMs.

To investigate interactions between sensory modality and emotional dimension of retrieval cues on memory retrieval and to bypass the problems that arise when using specific retrieval cues, we implemented the following study design to evoke olfactory and visually cued AMs. We prepared imagery instructions with adjectives that described an odor or picture to be imagined, but never mentioned a specific stimulus. The adjectives varied along the dimensions of valence and arousal. For example, the imagery instruction for a positively valenced highly arousing condition was: “Imagine a pleasant arousing odor/scene”. Thus, the odors and pictures that should be imagined were not predetermined but generated by the participants themselves. We expected this new method to have the following advantages: first, it uses an implicit approach and may, therefore, require less effort to carry out. Importantly, it produces memory-related imagery specific to that individual, which can thus be classified as an AM. Moreover, the cues include dimensions of valence/arousal, which should prompt memories with personal affective meaning.

The objective of the present study was a) to investigate the potential of odor imagery as a retrieval cue for AMs and b) to elucidate interactions between emotional dimensions (valence, arousal) and sensory modalities of memory cues, on the frequency, vividness, and age distribution (i.e., in which period of life the memories originated from), of the AMs reported. We hypothesized that the novel imagery induction approach would facilitate the retrieval of odor-related AMs, compared to classical deliberate cueing methods. Therefore, we assumed that the percentage of participants who would retrieve an AM would be higher than in the previous literature on AM retrieval after presentation of olfactory imagery (Willander & Larsson, 2008). Concerning the association between the retrieved memories and their age distribution, we hypothesized that more odor-imagery-cued AMs would originate in childhood than visual-imagery-cued AMs (e.g. Willander & Larsson, 2008). To control for age-related differences pertaining to the age distribution of the memories, we restricted the age of participants from 18 to 35 years. Concerning vividness, we expected that AMs retrieved after olfactory imagery would be rated as being more vivid, than as after visual imagery (Larsson et al., 2014). Based on the scarce previous findings regarding interactions between sensory modality and emotional dimensions of retrieval cues for AM (e.g. Knez et al., 2017), we hypothesized that frequency and vividness of odor-imagery-cued AMs would be higher in the negatively valenced conditions, whereas frequency and vividness of visual-imagery-cued AMs would be higher in the positively valenced conditions.

The sample was restricted to female participants, to control for sex effects concerning memory performance (Asperholm et al., 2019) and olfactory performance (Brand & Millot, 2001). Studies have found that women retrieve AMs faster than men, and rate the AMs as emotionally more intense compared to men (Andreano & Cahill, 2009; Seidlitz & Diener, 1998). Moreover, sex differences were reported for valence ratings of AMs. For example, Young et al. (2013) found that females recalled more negative and fewer positive AMs compared to males. Regarding olfactory performance, it is generally agreed upon that females show slightly superior olfactory abilities compared to men, such as heightened olfactory sensitivity, enhanced identification, and memory performance for odors (Brand & Millot, 2001; Wysocki & Gilbert, 1989).

## Method

### Sample

An a priori power analysis with G*Power 3.1.9.2 (Faul et al., 2007) indicated that a minimum sample size of *N* = 243 would be necessary to detect an effect size of *w* = 0.3 (i.e. medium effect) with a probability of 1 – *β* = 0.95, *α* = 0.05 for the *χ*^2^-tests to analyze AM frequency and a minimum sample size of *N* = 206 would be necessary to detect an effect size of part.*η*^2^ = 0.06 (i.e. medium effect) with a probability of 1 – *β* = 0.95, *α* = 0.05 for the main effects and interaction effects of sensory modality, valence and arousal of the imagery induction (2 × 2 × 2 ANOVAs) on AM vividness. Since previous literature suggests that not all participants would be able to generate mental images as well as AMs (e.g. Willander & Larsson, 2008), we recruited 296 females aged between 18 and 35 years (*M* = 23.48; SD = 3.89) to account for possible dropouts.The participation requirements (i.e., female sex and age < 18 > 35) were communicated prior to testing. Twentyeight male participants and nine participants aged > 35 filled out the survey anyway. They were excluded after testing. The participants were recruited via announcements at the university campus and through social media. 97% of the participants had a high school diploma, 86% were university students. Participants reported no mental health problems as indicated by the scores of the brief symptom inventory (BSI; Derogatis & Spencer, 1993), which screens for symptoms, such as insecurity, depression, somatization, anxiety, phobia, aggressiveness, paranoia, and psychoticism. We selected the BSI for this study because previous research has shown that psychological problems impact retrieval properties of mental images and autobiographical memories, such as emotionality (Walker et al., 2014), vividness (Zermatten et al., 2008), specificty (e.g., Barnhofer et al., 2002), and valence (Lyubomirsky et al., 1998). In the present sample, the T-score for the mean BSI score was in the normal range (T-score = 55). The study was performed in accordance with the Declaration of Helsinki and was approved by the ethics committee of the University of Graz. All participants gave written informed consent.

### Material and design

The participants were randomly allocated to one of the eight imagery groups (between-subject design see Table 1) to avoid carryover effects from one condition to the later condition. To evoke a mental image, we used standardized instructions (audio recordings; duration: 90 s) that differed concerning the sensory modality of the described stimulus [olfactory (O) vs. visual (V)], valence [pleasant (P) vs. unpleasant (UP)] and arousal [high (H) vs. low arousal (L)]. All descriptions for the imagery were abstract and never mentioned a specific stimulus. For example, the instruction stated: close your eyes and imagine that you are smelling an unpleasant arousing (pungent) odor. You can smell the odor clearly and it fills the area around you. For a more accurate perception of the unpleasant arousing odor, breathe in and out a few more times.

**Table 1:** Study design with eight imagery groups

Sensory modality	Valence	Arousal	*n*
Visual (V)	Unpleasant (UP)	Low arousal (L)	36
		High arousal (H)	36
	Pleasant (P)	Low arousal (L)	34
		High arousal (H)	40
Olfactory (O)	Unpleasant (UP)	Low arousal (L)	35
		High arousal (H)	36
	Pleasant (P)	Low arousal (L)	40
		High arousal (H)	39

The imagery task was followed by different ratings. The participants were asked to report, what specific mental image occurred (keywords). Additionally, they indicated whether or not the image referred to an event personally experienced in the past (i.e., autobiographical memory (AM); e.g., “The imagery reminded me of the smell when I once had to puke after a roller coaster ride”). We analyzed the percentage of participants who reported an AM image. If an AM image occurred, the participants reported their age, when the event had occurred. The events were either classified as childhood memories (age: 0–10 years), memories attributed to adolescence (age: 11–17 years), or more recent memories of adulthood (age: 18–35 years). Finally, the vividness of the imagery experience was rated on a seven-point Likert scale (0 = “no image at all “ to 6 = “very vivid image”). Reported arousal and valence for AM images were rated as a manipulation check (0 = “not arousing; very unpleasant” to 6 = “very arousing, pleasant”.

The groups did not differ concerning the number of participants and mean BSI score (all *p* > 0.05; for descriptives and statistics see supplementary Table 1).

### Procedure

The experiment was conducted via an online survey tool (Limesurvey GmbH, Hamburg). After obtaining written informed consent, the participants were asked for demographic data (age, sex, educational and occupational status). Then participants listened to the imagery instruction and named/rated the evoked AM images. Furthermore, participants filled out the BSI; (Derogatis & Spencer, 1993). Participants were asked to take part in the experiment in a quiet room.

### Statistical analysis

We compared the frequency of AM images (percentage of participants, who retrieved mental imagery with autobiographical content) between the factors Sensory Modality (visual vs. olfactory), Valence (pleasant vs. unpleasant), and Arousal (high vs. low arousal) of imagery induction via *χ*^2^-tests. Additionally, effects of Sensory Modality and Valence on the frequency of reported AMs for different periods of life (childhood, adolescence, adulthood) were examined via *χ*^2^-tests. (Arousal could not be analyzed due to low cell frequencies).

The effect of imagery induction (factors: Sensory Modality (visual vs. olfactory), Valence (pleasant vs. unpleasant), and Arousal (high vs. low arousal) on the reported vividness of AM imagery was tested using a 2 × 2 × 2 between subjects analysis of variance (ANOVA).

As exploratory analyses, the content of the reported AMs was classified according to five basic emotions: happiness (e.g., playing with a friend), sadness (e.g., funeral), anger (e.g., dispute with another person), anxiety (e.g. accident), and disgust (e.g., a person vomiting). The emotional content of the memories was classified by two independent psychologists with sufficient interrater agreement (Cohen’s Kappa = 0.61-0.81). The frequency of basic emotion-related content of AMs was compared between the conditions via *χ*^2^ tests.

Finally, as a manipulation check, the effects for imagery induction (factors: Sensory Modality (visual vs. olfactory), Valence (pleasant vs unpleasant), and Arousal (high vs low arousal) were tested on reported valence and arousal of AM imagery with a 2 × 2 × 2 ANOVA.

## Results

### Frequency of AM imagery

**Fig. 1:**
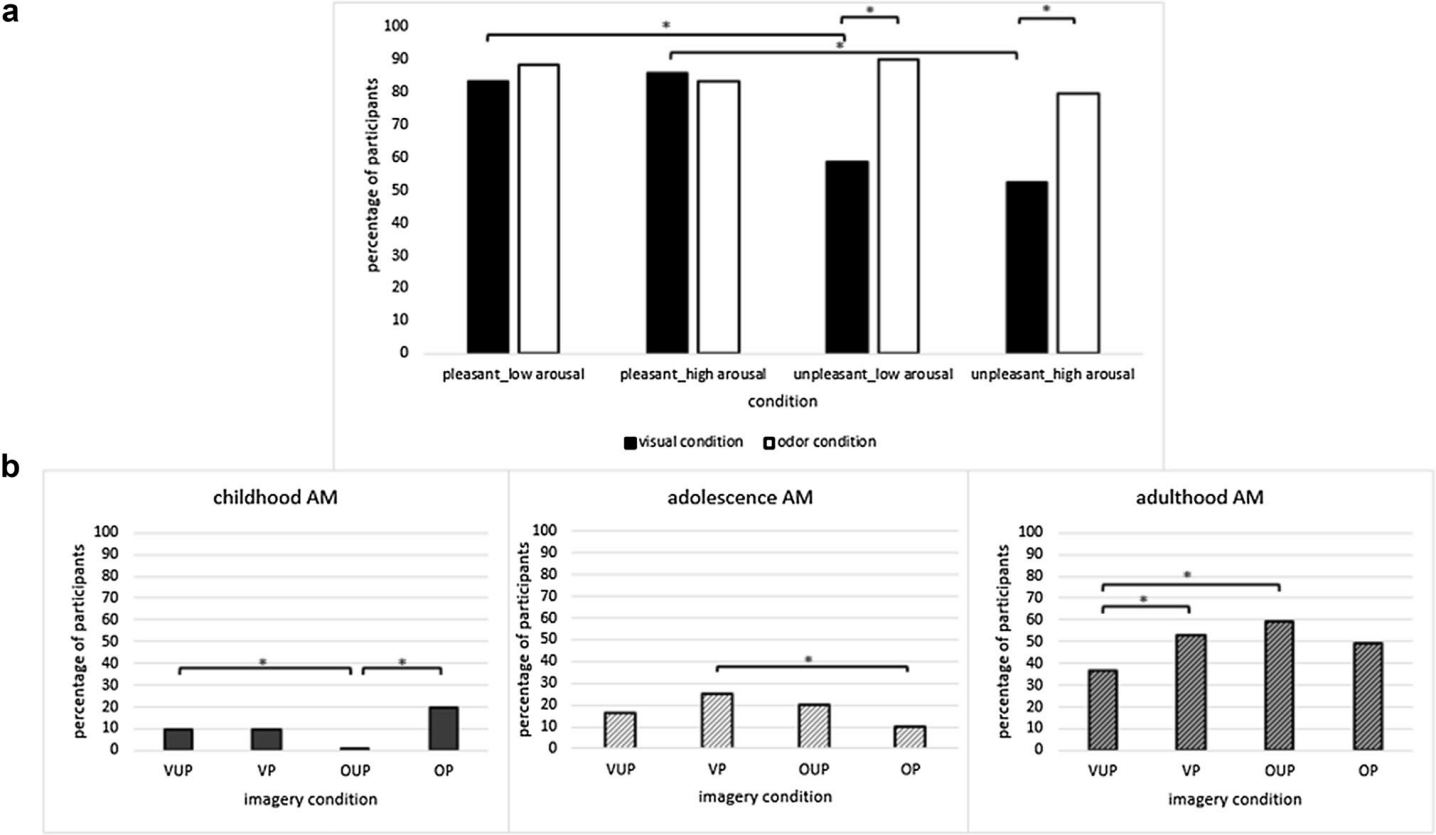
**a** Percentage of participants who reported an imagery experience with autobiographical content per imagery condition; **b** Percentage of participants who reported autobiographical memories related to age group (childhood-AM, adolescence-AM, adulthood-AM) and imagery condition. *VUP* visual unpleasant, *VP* visual pleasant, *OUP* olfactory unpleasant, *OP* olfactory pleasant. Asterisks indicate *p*-values < 0.05

A total of 230 participants (78%) reported an imagery experience with autobiographical content. Surprisingly, even more AM images were reported in the olfactory imagery condition (85%) compared to the visual imagery condition (70%; *χ*^2^ (1) = 10.22, *p* = 0.001, *V* = 0.186). Also in the pleasant imagery condition more participants reported an AM (85%) compared to the unpleasant imagery condition (71%; *χ*^2^ (1) = 9.25, *p* = 0.002, *V* = 0.177). Regarding interactions between sensory modality, valence, and arousal, the results of the *χ*^2^-analyses were in line with our hypothesis and showed that in the unpleasant conditions (with high and low arousal), more AM images were elicited via olfactory instructions (85%) compared to visual instructions (56%; *χ*^2^_High_ (1) = 6.39, *p* = 0.011, *V* = 0.284, *χ*^2^_Low_ (1) = 9.70, *p* = 0.002, *V* = 0.362). Furthermore, via visual imagery less AM images were evoked in the unpleasant (56%) compared to the pleasant imagery conditions (85%; *χ*^2^_High_ (1) = 9.91, *p* = 0.002, *V* = 0.361; *χ*^2^_Low_ (1) = 5.15, *p* = 0.023, *V* = 0.271). All other comparisons were not statistically significant (see Fig. 1a).

### Reported age distribution for AM imagery

Ten percent of participants retrieved AMs from their childhood, 18% reported AMs from adolescence and 50% reported AMs related to adulthood (22% did not report a life period).

- Percentage of childhood-related AMs per condition
  Regarding the frequency of childhood-related AMs, the conducted *χ*^2^-tests revealed results that contrasted our hypothesis on interaction effects between sensory modality and valence of memory cues. In the odor conditions, the frequency of reported childhood AMs differed between positively and negatively valenced imagery induction (*χ*^2^(1) = 14.15; *p* < 0.001; *V* = 0.307), whereas this was not the case in the visual conditions (*χ*^2^(1) = 0.003; *p* = 0.957; *V* = 0.004; VP = 9.72% vs VUP = 9.46%). In the odor conditions, childhood-related AMs were almost exclusively reported for pleasant imagery cues (20% of participants reported childhood-related AMs), whereas in the unpleasant conditions almost none of the reported AMs were childhood-related (0.01%). In the unpleasant condition more AMs related to childhood were reported in the visual (9%) compared to the odor imagery condition (0.01%; *χ*^2^(1) = 5.18; *p* = 0.023; *V* = 0.184); whereas in the pleasant condition the percentage of childhood-related AMs did not significantly (only marginally) differ between visual and odor imagery condition (*χ*^2^(1) = 2.85; *p* = 0.091; *V* = 0.141). See Fig. 1b).
- Percentage of adolescence-related AMs per condition
  In contrast to childhood-related AMs, the results on adolescence-related AMs and adulthood-related AMs are in line with our hypothesis. In the pleasant conditions, a higher percentage of participants reported AMs related to adolescence in the visual (25%) compared to the odor imagery conditions (10%; *χ*^2^(1) = 5.68; *p* = 0.017; *V* = 0.199). All other comparisons were not significant (all *p* > 0.078). See Fig. 1b).
- Percentage of adulthood-related AMs per condition
  In the unpleasant imagery conditions, more adulthood-related AMs were reported in the odor (60%) compared to the visual imagery condition (37%; *χ*^2^(1) = 8.10; *p* = 0.004; *V* = 0.230), whereas the percentage of participants who reported AMs related to adulthood did not differ between visual (53%) and odor imagery in the pleasant condition (49%; *χ*^2^(1) = 0.17; *p* = 0.677; *V* = 0.035). Furthermore, the percentage of participants who reported AMs related to adulthood was lower in the visual unpleasant (37%) compared to the visual pleasant imagery condition (53%, *χ*^2^(1) = 3.92; *p* = 0.048; *V* = 0.164), whereas the percentage of adulthood-related AMs in the odor condition did not significantly differ between pleasant and unpleasant imagery induction (*χ*^2^(1) = 1.57; *p* = 0.210; 0.102). See Fig. 1b).

### Vividness of AM imagery

The ANOVA revealed a significant main effect for Sensory Modality (see Table 2), however, in the contrasting direction of our hypothesis. Imagery was rated as more vivid in the visual conditions (*M* = 4.18, SD = 1.35) than in the olfactory imagery conditions (*M* = 3.66, SD = 1.34). All other main and interaction effects were not statistically significant; *p* > 0.173). For statistics, see Table 2; for means and standard deviations, see supplementary Fig. 1a.

**Table 2:** ANOVA statistics for the effects of imagery induction [factors: sensory modality (visual vs. olfactory), valence (pleasant vs unpleasant), and arousal (high vs low arousal)] on the reported vividness of AM imagery

Vividness of AM imagery	*F* (*df*)	*p*	Partial eta squared
Sensory Modality	7.16 (1, 222)	0.008	0.031
Valence	0.77 (1, 222)	0.382	0.003
Arousal	< 0.01 (1, 222)	0.954	< 0.001
Sensory Modality * valence	1.55 (1, 222)	0.215	0.013
Sensory Modality * arousal	1.45 (1, 222)	0.231	0.006
Valence * arousal	1.87 (1, 222)	0.173	0.008
Sensory Modality * valence * arousal	0.04 (1, 222)	0.841	< 0.001

### Content analysis

Overall, 85% of the reported AMs were classifiable to at least one of the basic emotions happiness, sadness, anger, anxiety, and/or disgust. Regarding differences between odor and visual imagery conditions, *χ*^2^-tests revealed that in the unpleasant imagery induction more sadness-, anxiety- and anger-related AMs were reported in the visual conditions, whereas more disgust-related AMs were reported in the odor conditions. In the pleasant conditions, 90% of reported AMs were happiness-related and percentages did not significantly differ between visual and odor imagery conditions (Table 3).

**Table 3:** Percentage of AMs related to basic emotions per condition

		%	*χ*^2^ (*df*)	*p*
*Happiness-related*				
Pleasant	Visual condition	93.44	1.48 (1)	0.224
	Odor conditions	86.88		
*Anger-related*				
Unpleasant	Visual condition	14.63	5.03 (1)	0.025
	Odor conditions	2.99		
*Sadness-related*				
Unpleasant	Visual condition	31.71	17.55 (1)	< 0.001
	Odor conditions	2.99		
*Anxiety-related*				
Unpleasant	Visual condition	26.83	16.53 (1)	< 0.001
	Odor conditions	1.49		
*Disgust-related*				
Unpleasant	Visual condition	0.02	64.64 (1)	< 0.001
	Odor conditions	82.09		

### Manipulation check: valence/arousal of AM imagery

As expected, the ANOVA identified a significant main effect for valence of imagery instruction on the valence ratings for the AM imagery (supplementary Table 2). AMs retrieved in the pleasant imagery conditions were rated as more pleasant (*M* = 5.33, SD = 1.19) than AMs retrieved in the unpleasant conditions (*M* = 2.25, SD = 1.52). All other effects were not significant (all *p* > 0.12).

However, the computed ANOVA for arousal ratings was only partly in line with our instructions. The results revealed significant interactions between Sensory Modality, Valence, and Arousal of imagery instruction (see supplementary Table 2). In the visual imagery condition with unpleasant/high arousal adjectives, AMs were rated more arousing than in the visual imagery condition with unpleasant/low arousal adjectives; *F*(1,217) = 8.67, *p* = 0.004, part*η*^2^ = 0.038). All other effects were nonsignificant (*p* > 0.345). For means and standard deviations see supplementary Fig. 1b and c; for statistics see supplementary Table 2.

## Discussion

This study investigated the effects of sensory modalities (olfaction, vision) and emotional dimensions (valence, arousal) of imagery cues on the frequency, vividness, and age distribution of reported AMs. As a new approach, we used imagery instructions as retrieval cues and not specific odors or pictures (e.g., Bruijn & Bender, 2018; Chu & Downes, 2002; Jellinek, 2004; Rubin et al., 1984). Moreover, we described the to-be-imagined odors/scenes with adjectives (e.g., “imagine a pleasant arousing odor”) and never mentioned a specific stimulus or situation.

This new approach for AM retrieval was very successful in terms of frequency of participants retrieving an AM. The majority of participants (78%) were able to generate a mental image with autobiographical content. In contrast, Willander & Larsson (2008), who used specific to-be-imagined odors to prompt AM (e.g.”imagine the smell of a rose”), observed an AM retrieval rate of only 40%. Previous research consistently revealed that odors were less effective to elicit AMs than visual cues (e.g., Larsson et al., 2014; Rubin et al., 1984; Stevenson & Case, 2005; Willander & Larsson, 2007). In the present study, this disadvantage for olfactory cues was not present. A possible reason for previous literature reporting odor-related experiences to be retrieved less frequently than experiences related to visual stimuli might be their use of specific stimuli for AM cueing (i.e., odors or pictures of the same object (e.g., lily). Since we rely on vision as our primary sense, the visual modality might be prioritized when using specific stimuli to retrieve AMs, which possibly restricts access to odor-related experiences. In contrast to stimuli of other sensory modalities, odors are more often perceived unconsciously (Sela & Sobel, 2010). We speculate that due to the opportunity to freely generate individual mental imagery based on more implicit inductions used in our study, the visual modality did not take precedence and therefore facilitated retrieval of odor-related AMs.

In terms of the reported age distribution of AMs, most memories were attributed to adulthood/adolescence, whereas only a small percentage of participants (approximately 10%) retrieved childhood-related memories. This is in line with previous research on the reminiscence bump [i.e., the tendency to access more personal memories from young adulthood than from other periods of life (Munawar et al., 2018)]. Regarding interactions between sensory modalities and emotional dimensions of imagery instructions on reported period of life for AMs, our findings are only partly in line with previous research and our hypothesis stating that odor cues are particularly efficient in cueing old childhood memories and unpleasant memories (e.g., Knez et al., 2017; Larsson et al., 2014). The results showed that unpleasant odor imagery cues were associated with more unpleasant memories (approximately 60%) for adulthood than visual imagery cues (approximately 37%). Childhood memories, however, were almost exclusively retrieved when using pleasant olfactory imagery cues (approximately 20%).

This interaction effect might be explained by our findings in the exploratory content analysis: here, we showed that AMs in the odor-imagery condition were predominantly disgust- and happiness-related. Visual imagery-cued AMs were associated with diverse emotions, such as happiness, fear, sadness, and anger but not with disgust. This finding is in line with previous research (Bensafi et al., 2002), that found that specific emotions (e.g., happiness, sadness, anger, fear, disgust) are not equally evoked by odors, so that disgust and happiness were significantly more frequent than other emotions. An explanation for this could be provided by the hierarchy of senses in humans. In most areas of functioning, humans rely on the sense of vision (e.g., San Roque et al., 2015), whereas the sense of smell is important only for specific areas, e.g. disgust-related threats such as spoiled food, body excretions; (Stevenson et al., 2010). These threats can be effectively detected with the chemical senses (taste and olfaction). Over and above the emotional dimensions valence and arousal, the emotion category disgust appears to have a special salience in memory relative to other emotion categories (Chapman et al., 2013; Schienle et al., 2021). The strong link between disgust and olfaction and the memory bias for disgust stimuli might be an explanation for the higher percentage of participants reporting AMs in the unpleasant odor imagery condition relative to the unpleasant visual imagery condition. It is known, that olfactory performance (e.g., Cameron, 2018), as well as disgust responses (e.g. Rottman, 2014), change across the lifespan. Disgust responses are less pronounced in early childhood and increase with age. Therefore, it is not surprising that in the unpleasant odor imagery conditions more AMs were reported for adulthood compared to childhood (Rottman, 2014).

In terms of quality, contrary to our hypothesis, AM imagery was rated as less vivid in the olfactory compared to the visual condition. Similar findings have been reported by Arshamian et al. (2013) and Arshamian and Larsson (2014) who observed that olfactory imagery is typically experienced as less vivid than imagery generated from other modalities. However, in their review, Larsson et al. (2014) concluded that odor-cued AMs are more vivid than AMs cued via other sensory cues. In line with this view, Willander and Larsson (2008) stated that the perception of a real odor is a prerequisite for a vivid AM. It has to be acknowledged, however, that the average difference of 0.52 points (on a seven point Likert scale) in vividness ratings between olfactory and visually cued AMs is indeed statistically significant, but might not be relevant in practical application.

Possible implications of our findings are: imagery is an easy-to-use method, where no additional material is needed. First, this opens up new possibilities in research. Through the novel imagery approach data can be collected remotely in large online samples, which might help to accelerate this line of research. Second, imagery could complement existing methods of psychological practice. Positively valenced odor imagery to cue pleasant childhood memories could be used in resource activation in clients who have difficulties accessing positive autobiographical memory narratives. Furthermore, negatively valenced odor imagery could be used to access recent disgust-related memories. Such memories are relevant in the context of posttraumatic stress disorder (PTSD). Our new approach of nonspecific induction of olfactory imagery to access AM might be an option as one component of exposure therapy, which so far relied on the administration of real odors (Vermetten & Bremner, 2003).

However, we need to acknowledge the following limitations of the present investigation. Our study included an exclusively female sample with a restricted age range and educational status (mainly university students). Thus, our results cannot be generalized to other groups. Future studies on sex and age differences in the imagery task are strongly recommended. Further shortcomings of the present investigation concern the imagery instruction. The valence instructions for the imagery worked well; AMs retrieved after pleasant retrieval cues were rated as more pleasant than AMs retrieved after unpleasant retrieval cues. However, descriptions of stimuli inducing high vs. low arousal did not lead to AMs that differed in experienced arousal. Similar findings regarding the arousal dimension have been reported by Schulkind and Woldorf (2005), Sheldon and Donahue (2017) and Walker et al. (2003). Therefore, future studies need to investigate evoked arousal by imagery instructions more carefully. Moreover, it would be important for future research on imagery as a retrieval cue for AMs to collect additional information (for review see Sutin & Robins, 2007), such as the level of specificity of reported AMs. For example, Conway and Pleydell-Pearce (2000) suggested to classify AMs according to three specificity levels (i.e., unique, specific events; repeated, general events; and life periods). We instructed participants to refer to specific events that were personally experienced (i.e., unique, specific events). However, it would be interesting to additionally assess the level of AM specificity as a manipulation check. Furthermore, information on effort needed for AM retrieval, and richness of elaborated details would be important to assess in future research. This is particularly interesting with regard to valence of the imagery induction, as previous research indicated that spontaneous memories are more often triggered by unpleasant cues (e.g., Schlagman & Kvavilashvili, 2008) and memories that were initially accessed to high-arousing cues were later described with more detail (Sheldon et al., 2020).

To the best of our knowledge, this is the first study to investigate the effects of sensory modalities and emotional dimensions of imagery as retrieval cues on AMs. Future studies need to replicate the present findings and broaden the research scope. For example, it would be interesting to investigate odor imagery as a retrieval cue in clinical samples with diagnoses of depression, or PTSD.

## Supplementary Information

Below is the link to the electronic supplementary material.

Supplementary file1 (PDF 127 KB)

## Data Availability

The raw data supporting the conclusions of this article will be made available by the authors, without undue reservation.
