# Supplementary material for: Olfactory imagery as a retrieval method for autobiographical memories
Source: Psychol Res. 2022 Jul 5;87(3):862–71. doi: 10.1007/s00426-022-01701-y (PMC10017607; doi:10.1007/s00426-022-01701-y)
Supplement: Supplementary file 1 — Supplementary file1 (PDF 127 KB) [file 426_2022_1701_MOESM1_ESM.pdf]

Supplementary Table 1. Sample descriptives (number of participants, self-ratings of olfactory performance, and mental well being (BSI_sum) per condition)

		condition	***N***		***Chi2 (df)***		***p***	
**number**	**visual**	PL	36		1.03(7)		.994	
		PH	36					
		UPL	34					
		UPH	40					
	**odor**	PL	35					
		PH	36					
		UPL	40					
		UPH	39					

			M	***SD***	***F(df)***		***p***	***Part.η^2***
**Olf. perf (self-rating)**	**visual**	PL	4.67	.93	Sense category	.539(1,288)	.463	.002
		PH	4.31	1.37	Valence	.023(1,288)	.880	<.001
		UPL	4.32	1.36	Arousal	.169(1,288)	.681	.001
		UPH	4.50	1.36	Sense cat x valence	.126(1,288)	.723	<.001
	**odor**	PL	4.49	1.12	Sense cat x arousal	.047(1,288)	.828	<.001
		PH	4.17	1.38	Valence x arousal	3.632(1,288)	.058	.012
		UPL	4.22	1.39	Sense cat. x val.x arous.	.006(1,288)	.941	.000
		UPH	4.49	1.07				
**BSI_sum**	**visual**	PL	38.17	30.24	Sense category	.003(1,286)	.959	<.001
		PH	39.31	29.19	Valence	.002(1,286)	.961	<.001
		UPL	31.97	26.40	Arousal	1.12(1,286)	.291	.004
		UPH	41.78	28.04	Sense cat x valence	.250(1,286)	.617	.001
	**odor**	PL	32.06	26.82	Sense cat x arousal	.306(1,286)	.581	.001
		PH	41.67	35.87	Valence x arousal	.275(1,286)	.617	.001
		UPL	41.49	29.28	Sense cat. x val.x arous.	3.24(1,286)	.073	.011
		UPH	35.31	25.52				

Supplementary Table 2. ANOVA statistics for the manipulation check

	F(df)	*p*	*Part.*η^2
**Valence of AM**			
Sensory category	.62 (1, 206)	.431	.003
valence	252.00 (1, 206)	<.001	.550
arousal	.01 (1, 206)	.940	<.001
Sensory category * valence	2.44 (1, 206)	.119	.012
Sensory category * arousal	<.01 (1, 206)	.994	<.001
valence * arousal	.28 (1, 206)	.600	.001
Sensory category * valence * arousal	1.33 (1, 206)	.250	.006
**Arousal of AM**			
Sensory category	5.45 (1, 217)	.020	.025
valence	25.06 (1, 217)	<.001	.104
arousal	.60 (1, 217)	.440	.003
Sensory category * valence	.20 (1, 217)	.652	.001
Sensory category * arousal	3.69 (1, 217)	.056	.017
valence * arousal	3.36 (1, 217)	.068	.015
Sensory category * valence * arousal	5.76 (1, 217)	.017	.026

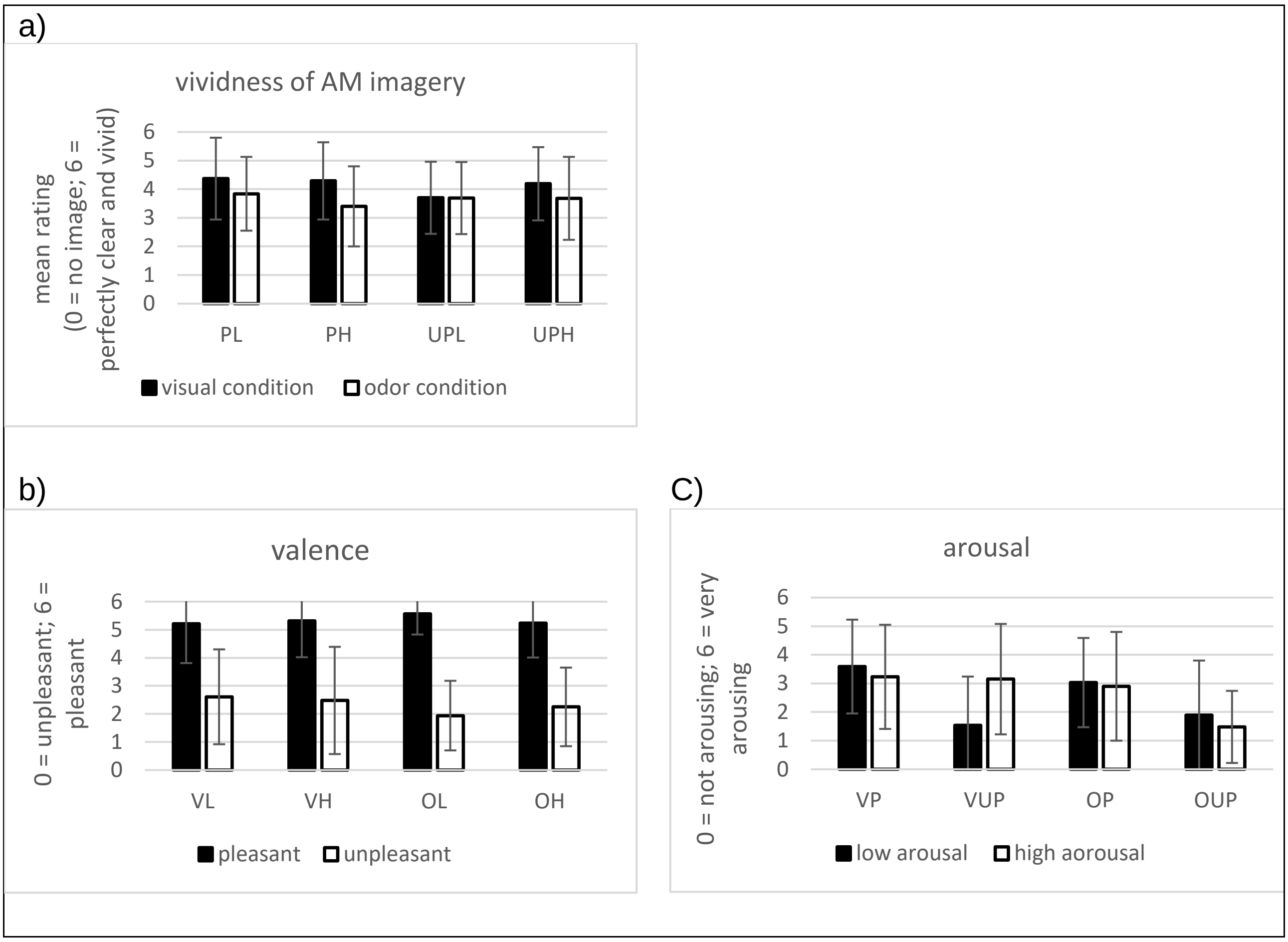

Supplementary Fig 1. Mean ratings of the a) vividness, b) valence and c) arousal of AM imagery

Footnote: AM=autobiographical memory, V=visual imagery condition, O=odor imagery condition, P=pleasant condition, UP=unpleasant condition, L=low arousal condition, H=high arousal condition
